# Distribution of Intracranial Major Artery Stenosis/Occlusion According to RNF213 Polymorphisms

**DOI:** 10.3390/ijms21061956

**Published:** 2020-03-13

**Authors:** Jinkwon Kim, Young Seok Park, Min-Hee Woo, Hui Jeong An, Jung Oh Kim, Han Sung Park, Chang Soo Ryu, Ok Joon Kim, Nam Keun Kim

**Affiliations:** 1 Department of Neurology, Yongin Severance Hospital, Yonsei University College of Medicine, Yongin 16995, Korea; antithrombus@gmail.com; 2 Department of Neurology, CHA Bundang Medical Center, CHA University, Seongnam 13496, Korea; RA3520@chamc.co.kr; 3 Department of Neurosurgery, Chungbuk National University Hospital, Chungbuk National University, College of Medicine, Cheongju 28644, Korea; youngseokparkmd@gmail.com; 4 Department of Biomedical Science, College of Life Science, CHA University, Seongnam 13488, Korea; tody2209@naver.com (H.J.A.); jokim8505@gmail.com (J.O.K.); hahnsung@naver.com (H.S.P.); regis2040@nate.com (C.S.R.)

**Keywords:** intracranial major artery stenosis/occlusion, RNF-213, moyamoya disease, single nucleotide polymorphism

## Abstract

Intracranial major artery stenosis/occlusion (ICASO) is the major cause of ischemic stroke. Recent studies have suggested that variants of *RNF213*, a susceptibility gene for moyamoya disease (MMD), are also related to non-MMD ICASO. Regarding the predominant involvement of steno-occlusion on anterior circulation in MMD, we hypothesized that the ICASO distribution pattern (anterior/posterior) in non-MMD may differ according to *RNF213* variants. This study analyzed 1024 consecutive Korean subjects without MMD who underwent computed tomography angiography (CTA) or magnetic resonance angiography (MRA). We evaluated four single nucleotide polymorphisms (SNPs) in the exon region of *RNF213*: 4448G > A (rs148731719), 4810G > A (rs112735431), 4863G > A (rs760732823), and 4950G > A (rs371441113). Associations between *RNF213* variants and anterior/posterior ICASO were examined using multivariate logistic regression analysis. Anterior ICASO was present in 23.0% of study subjects, and posterior ICASO was present in 8.2%. The GA genotype of *RNF213* 4810G > A (adjusted odds ratio (AOR) [95% confidence interval (CI)], 2.39 [1.14–4.87] compared to GG; *p* = 0.018) and GA genotype of *RNF213* 4950G > A (AOR [95% CI], 1.71 [1.11–2.63] compared to GG; *p* = 0.015) were more frequent in subjects with anterior ICASO. The genotype frequency of *RNF213* 4863G > A differed significantly according to the presence of posterior ICASO. Further investigations of the functional and biological roles of *RNF213* will improve our understanding of the pathomechanisms of ICASO and cerebrovascular disease.

## 1. Introduction

Intracranial major artery stenosis/occlusion (ICASO) is the major cause of ischemic stroke [1,2]. ICASO can result from atherosclerosis, thromboembolism, or non-atherosclerotic vascular disease, such as dissection, vasculitis, or moyamoya disease (MMD) [3]. Really interesting new gene (RING) finger protein 213 (RNF213) is encoded by the *RNF213* gene (DDBJ/EMBL/GenBank accession number AB537889) on chromosome 17q25, which has received considerable attention as a susceptibility gene for MMD and ICASO in Asians [4,5,6]. The functional role of RNF213 on cerebral vasculature has not been established, but experimental studies have suggested that it affects cerebral blood perfusion, angiogenesis, and oxygen consumption [7,8,9,10].

ICASO can be present in anterior and posterior cerebral circulation. It has been known that anterior and posterior ICASO differs in clinical characteristics, risk factors, histopathology, and genetic susceptibility [11,12,13]. *RNF213* is recognized as the major susceptibility gene for MMD, which is typically characterized by progressive steno-occlusive changes in the terminal part of the internal carotid arteries and/or their branches (anterior circulation) [14,15]. Regarding the potential genetic contributions to ICASO, we hypothesized that the distribution of anterior/posterior ICASO in non-MMD may also differ according to genetic variants of *RNF213*. To test our hypothesis, we assessed the association between four single nucleotide polymorphisms (SNPs) in the exon region of *RNF213* (4448G > A, 4810G > A, 4863G > A, and 4950G > A) and the distribution pattern of ICASO in non-MMD Koreans.

## 2. Results

### 2.1. Characteristics

This study included 1024 non-MMD subjects (636 ischemic stroke and 388 non-stroke subjects) who underwent genotyping of *RNF213* variants and angiographic evaluation. Their mean age was 63.6 ± 11.4 years, and 47.0% were male. Baseline characteristics of the study subjects are shown in Table 1. ICASO was present in 27.6%. Compared with subjects without ICASO, those with ICASO were more likely to be older and have hypertension, diabetes mellitus (DM), and ischemic stroke. Regarding the distribution of ICASO, 23.0% of subjects had anterior ICASO, and 8.2% had posterior ICASO.

### 2.2. RNF213 Genotypes

Table 2 shows the genotype frequencies of the *RNF213* variants. For *RNF213* 4448G > A, the frequency of minor allele (A) was 7.7%. The minor allele (A) frequencies of 4810G > A, 4863G > A, and 4950G > A were 2.0%, 11.7%, and 7.0%, respectively. Carrier frequencies of at least one A allele (minor allele) for the four SNPs were 14.9%, 4.0%, 22.2%, and 13.6%. The distribution of *RNF213* polymorphisms satisfied the Hardy–Weinberg equilibrium.

### 2.3. Relationship between RNF213 Genotypes and ICASO

Table 3 shows the genotype distribution of *RNF213* variants according to the presence of ICASO.

We investigated the relationships between distributions of the four *RNF213* variants and the presence of ICASO using multivariate logistic regression analysis (Table 4). Patients with ICASO were more likely to have the *RNF213* 4810 (rs112735431) GA genotype than the GG genotype, but the association was not statistically significant (adjusted odds ratio (AOR) [95% confidence intervals (CI)], 2.05 [0.98–4.21]; *p* = 0.053). None of the other *RNF213* variants were associated with the presence of ICASO.

### 2.4. Relationship between RNF213 Genotypes and Anterior/Posterior ICASO

When we evaluated the relationship between *RNF213* variants and the distribution of ICASO (anterior/posterior) using the multivariate logistic regressions (Table 4), anterior ICASO was significantly associated with the *RNF213* 4810 (rs112735431) GA genotype (AOR [95% CI], 2.39 [1.14–4.87]; *p* = 0.018) compared with the GG genotype. Anterior ICASO was also significantly more likely with the *RNF213* 4950 (rs371441113) GA genotype (AOR [95% CI], 1.71 [1.11–2.63]; *p* = 0.015) compared with the GG genotype. In the analysis regarding the posterior ICASO, the presence of posterior ICASO was significantly associated with the *RNF213* 4863 (rs760732823) genotype. Compared with the *RNF213* 4863 GG genotype, the GA genotype was associated with lower likelihood of posterior ICASO (AOR [95% CI], 0.29 [0.11–0.64]; *p* = 0.005), but the AA genotype was associated with higher odds of posterior ICASO (AOR [95% CI], 8.55 [2.17–32.51]; *p* = 0.002).

### 2.5. Haplotype Analysis

**Figure 1:**
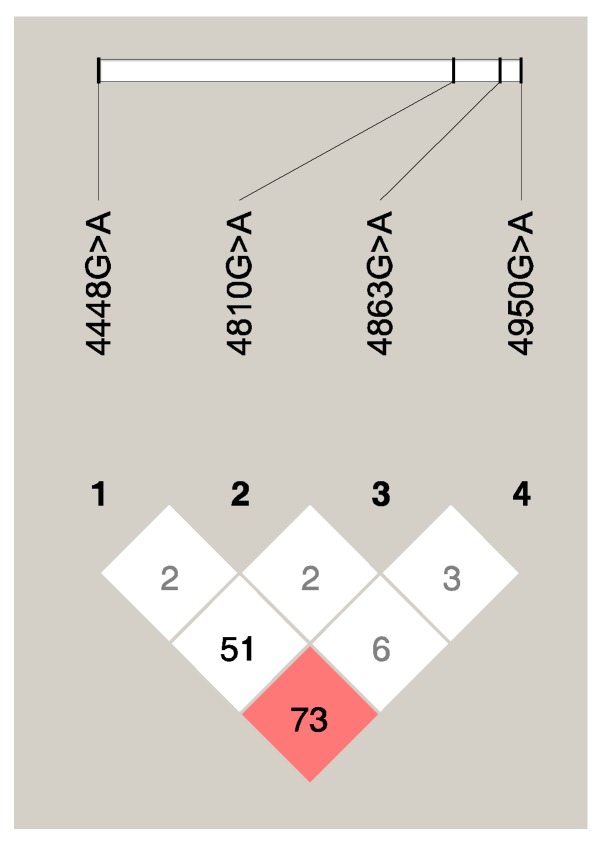
Linkage disequilibrium plot of four RNF213 single nucleotide polymorphisms. The linkage disequilibrium between each single nucleotide polymorphism (SNP) is measured as the D′ statistic (×100), shown in the diamond at the intersection of the diagonals of the two corresponding SNPs. Strong linkage disequilibrium is represented in red, and weak linkage disequilibrium is represented in white.

We evaluated the linkage disequilibrium of the *RNF213* variants (4448G > A (rs148731719)/4810G > A (rs112735431)/4863G > A (rs760732823)/4950G > A (rs371441113)) using the Haploview program (Figure 1). Among the four SNPs, there was a strong linkage disequilibrium (0.73 of D′ statistic and 0.480 of *r*^2^) between loci 4448G > A (rs148731719) and 4950G > A (rs371441113). Other combinations of *RNF213* SNPs did not exhibit strong linkage disequilibrium.

We performed haplotype analyses of the four SNPs for the presence of ICASO, anterior ICASO, and posterior ICASO (Table 5). In the haplotype analyses, GAGG (4448G/4810A/4863G/4950G) was associated with a significantly increased risk of ICASO and anterior ICASO. Other combinations did not show significant association with ICASO distribution.

## 3. Discussion

In the current study, we evaluated the distribution of four polymorphisms in the exon sequence of *RNF213*, the MMD susceptibility gene, and the relationship between these polymorphisms and ICASO in non-MMD Koreans. We found that *RNF213* 4810G > A (rs112735431) and 4950G > A (rs371441113) were significantly associated with the presence of anterior ICASO, whereas *RNF213* 4863G > A (rs760732823) was associated with posterior ICASO. If further studies validate the relationship between the *RNF213* variants and anterior/posterior ICASO, detection of *RNF213* variants could be applicable to a screening method for ICASO or prediction of disease prognosis. Discovery of a pathogenic role of RNF213 gene in ICASO may provide a novel therapeutic target in ICASO with high risk for stroke.

The *RNF213* gene encodes a RING finger protein that possesses both ubiquitin ligase activity and ATPases associated with diverse cellular activities’ (AAA+) ATPase activity [16]. Although *RNF213* variants have been associated with various vascular disorders, the mechanisms through which they contribute to these disorders are unknown [17]. Experimental reports have suggested multiple pathogenic mechanisms involving endothelial function, smooth muscle cell proliferation, inflammatory signaling pathways, hemostasis, angiogenesis, vascular remodeling, and response to hypoxia [7,9,10,18,19,20,21,22]. However, an *RNF213*-deficient mouse model did not exhibit any vascular abnormality [23]. Currently, the exact biochemical and pathologic roles of RNF213 in vascular disorders remain controversial.

Beyond conflicting reports regarding the functional role of RNF213, epidemiologic data have shown significant associations between *RNF213* variants and intracranial vascular disorders including MMD, ICASO, cerebral artery dissection, and intracranial aneurysm [4,24,25,26]. At least 24 genetic changes in *RNF213* have been associated with MMD [21]. The *RNF213* 4810G > A (rs112735431) is considered a major genetic risk factor for MMD; the carrier rate of this SNP in East Asians with MMD is >70% [18,27,28]. Although the *RNF213* 4810G > A variant has never been detected in Caucasians, there are rare missense *RNF213* variants, which have been associated with MMD in Caucasians, especially those with childhood-onset or familial disease [29,30]. Beyond the association with MMD, a number of studies have reported frequent *RNF213* variants in patients with non-MMD ICASO [2,31,32]. In a meta-analysis of 11 studies including 1778 ICASO patients and 3140 controls in East Asians, *RNF213* 4810G > A was significantly associated with an increased risk of ICASO [17]. High-resolution magnetic resonance angiography (MRA) showed that ICASO with *RNF213* 4810G > A was associated with a negative remodeling pattern, which is a hallmark of MMD, in contrast to the positive remodeling pattern of classical atherosclerotic ICASO [33]. In addition to the relationship with the presence of intracranial cerebrovascular disease, *RNF213* variant genotypes may also be associated with the disease progression and long-term outcome. Among patients with MMD, the homozygous variant of *RNF213* 4810G > A was associated with an increased likelihood of onset at a younger age, cerebral infarction at diagnosis, and cognitive impairment during long-term follow-up [34]. In a study of 59 relatives of patients with MMD, *RNF213* 4810G > A heterozygous carriers had a higher risk of developing ICASO during angiographic follow-up [35]. The presence of *RNF213* 4810G > A variant was at increased risk for ischemic stroke, which was largely attributable to large-artery atherosclerosis [36]. Recent reports have also described associations between *RNF213* variants and extracranial systemic vasculopathy involving coronary, renal, and pulmonary arteries [37,38,39].

Because of the predominant involvement of the anterior cerebral circulation in MMD, we hypothesized that the distribution pattern of ICASO may differ according to *RNF213* variants. In our results, we found that *RNF213* 4810G > A (rs112735431) and 4950G > A (rs371441113) increased the likelihood of anterior ICASO. Both of these variants have been reported to be significantly associated with MMD in Korean and Chinese populations [18,21,40,41]. Similarly, there was a study of 221 Japanese patients, which found that *RNF213* 4810G > A was significantly associated with anterior ICASO but not posterior ICAS [42]. In the case-control study with a total of 46,958 Japanese people, *RNF213* 4810G > A variant carriers more frequently had intracranial anterior circulation stenosis than non-carriers (60.0% versus 27.3%, *p* = 0.004) [36]. Another study of 70 early-onset stroke patients with ICASO in Japan found that 17 *RNF213* 4810G > A carriers had anterior ICASO, whereas only 1 carrier of this variant had posterior ICASO as well, and the *RNF213* 4810G > A variant was more common in patients with MCA or ACA stenosis (17/44) than in patients with posterior ICASO (1/11) [43]. However, there have also been negative reports indicating that the distribution of anterior and posterior ICASO did not differ between *RNF213* variant carriers and non-carriers [2,32]. The inconsistent results between studies may be attributed to differences in study populations; the two negative reports included only ICASO (+) subjects and compared the distribution of anterior and posterior ICASO according to *RNF213* variants. Because of our limited understanding of the pathogenetic role of RNF213 and insufficient clinical data, it is difficult to make conclusions regarding the relationships between *RNF213* variants and distribution patterns of ICASO. However, predominant involvement of the anterior cerebral circulation in carriers with *RNF213* variants of 4810G > A and 4950G > A suggests an inter-relationship between ICASO and MMD and the possibility of a shared genetic pathomechanism.

In the current study, the presence of posterior ICASO differed significantly according to the genotype distribution of *RNF213* 4863G > A (rs760732823). As underlying risk factors and genetic predisposition differ between anterior and posterior ICASO, *RNF213* variants may have divergent effects on the anterior and posterior cerebral circulations [13]. To our knowledge, no prior report has shown a significant relationship between *RNF213* variants and posterior ICASO. Interestingly, compared with the GG wild type of 4863G > A (rs760732823), the GA genotype was associated with a lower risk of posterior ICASO, but the AA genotype was associated with a higher risk. Compared with *RNF213* 4810 (rs112735431) and 4950 (rs371441113), which are both susceptibility genes for MMD, little is known about *RNF213* 4863G > A (rs760732823). We previously evaluated the distribution of *RNF213* 4863G > A between Korean MMD patients and controls; there was no significant difference according to *RNF213* 4863G > A [21]. More research is required to verify whether the relationship between *RNF213* 4863G > A and posterior ICASO is duplicated in other study populations.

We acknowledge potential limitations of this study. Although many studies have reported associations between *RNF213* variants and cerebrovascular disorders, the underlying pathogenic process has not been established. Without understanding the pathogenetic mechanisms, our data from case-control design could not access the causal relationship between *RNF213* variants and ICASO. Furthermore, ICASO and MMD, both *RNF213*-related disorders, are progressive cerebrovascular diseases [35,44]. Because our study lacked follow-up data, we did not provide information about the progression of ICASO or the prognostic value of *RNF213* variants. ICASO is a heterogeneous disease, influenced by various environmental and genetic factors [2,45]. Although atherosclerotic stenosis of a major intracranial artery is the most common type of ICASO, different etiologies and risk factors can influence the development of ICASO [4,31]. We adjusted for multiple conventional vascular risk factors, but the possibility of unknown potential confounding factors remains. Although we excluded MMD patients based on angiographic findings, it is possible that patients with early changes or atypical patterns of MMD might have been included, which may have contributed to the association between ICASO and *RNF213* variants [17,31,33]. In addition, the study was performed at a single hospital, and a large portion of the study participants had ischemic stroke. Thus, the distribution of *RNF213* variants in our subjects may have differed from that in the general population of Koreans. Further analysis according to the degree of ICASO was lacking in the current study. Use of statins, antiplatelets, and anti-hypertensive agents may influence the development and progression of ICASO. Unfortunately, due to the limitations of the retrospective design, we could not collect the data for medications. As the pathogenesis of MMD is known to be ethnically diverse, the effect of *RNF213* variants on ICASO may have varied in other populations [26]. Moreover, the low prevalence of *RNF213* variants and the small sample size of this study may have limited the statistical power to detect differences between subgroups and gene–environmental interactions. We assessed four SNPs in the current study, but there are other potential susceptibility variants in *RNF213*. To overcome the potential limitations and to improve our understanding of the influence of *RNF213* on ICASO, a wide range of research is required to explore the genetic pathogenesis and epidemiology of this common cerebrovascular disorder.

## 4. Methods

### 4.1. Study Subjects

**Figure 2:**
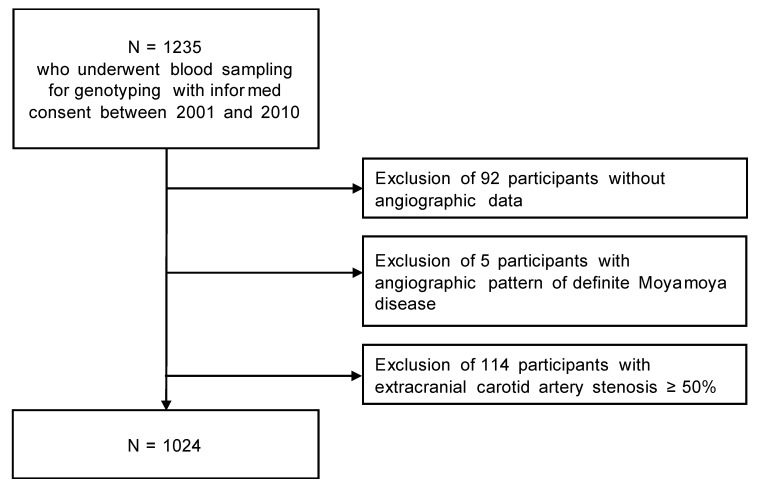
Flowchart of study participant inclusion and exclusion.

Study subjects were consecutively recruited from the Department of Neurology at CHA Bundang Medical Center, CHA University (an 800-bed teaching hospital in Gyeonggi-do, South Korea), between 2001 and 2010 (Figure 2). Subjects were selected from those who underwent blood sampling for genotyping and cerebral angiographic evaluation by MRA or computed tomography angiography (CTA). Patients with proximal carotid artery occlusion were excluded because the proximal carotid occlusion often does not allow the evaluation of intracranial arteries. Individuals who satisfied the criteria for definite MMD were excluded from the study [43,46]. The medical records were reviewed to identify the presence of hypertension, DM, hyperlipidemia, atrial fibrillation, or ischemic stroke. Hypertension was defined as use of antihypertensive medication or a resting systolic blood pressure ≥140 mmHg or resting diastolic blood pressure ≥90 mmHg on repeated measurements. DM was defined as use of antidiabetic medications or a fasting plasma glucose ≥7.0 mmol/L or glycosylated hemoglobin ≥6.5%. Hypercholesterolemia was defined as use of lipid-lowering agents or a low-density lipoprotein cholesterol ≥4.1 mmol/L or total cholesterol ≥6.2 mmol/L. Ischemic stroke was defined as acute neurological dysfunction of vascular origin, confirmed by brain magnetic resonance imaging or computed tomography. All participants provided written informed consent. The study was approved by the Institutional Review Board of CHA Bundang Medical Center (BD2012-136D).

### 4.2. Distribution of ICASO

Angiographic data (MRA or CTA) of the intracranial cerebral arteries were evaluated by two neurologists (J.K., M.H.W.). ICASO was defined as occlusion or ≥50% stenosis in the intracranial portion of internal carotid artery (ICA), middle cerebral artery (MCA), anterior cerebral artery (ACA), posterior cerebral artery (PCA), vertebral artery (VA), or basilar artery (BA). The degree of stenosis in each intracranial cerebral artery was measured using the Warfarin vs. Aspirin for Symptomatic Intracranial Disease method [47]. According to the intracranial angiographic findings, the presence of “anterior ICASO” (involving the ICA, ACA, or MCA) and “posterior ICASO” (involving the PCA, VA, or BA) was recorded.

### 4.3. Genotyping of RNF213 Variants

In our prior study, we identified four SNPs in the exon region of *RNF213*: 4448G > A (rs148731719), 4810G > A (rs112735431), 4863G > A (rs760732823), and 4950G > A (rs371441113) based on prior reports in Asians and a literature review [21]. Information regarding the SNPs was obtained from the HapMap database (http://hapmap.ncbi.nlm.nih.gov/). For genotyping of these SNPs, DNA was extracted from peripheral blood samples using the G-DEX blood extraction kit (iNtRON Biotechnology, Inc., Seongnam, Korea) according to the manufacturer’s instructions. *RNF213* variants were detected by polymerase chain reaction–restriction fragment length polymorphism (PCR–RFLP) analysis. *RNF213* 4810G > A (rs112735431) was evaluated in all study subjects (*n* = 1024), whereas 4448G > A (rs148731719), 4863G > A (rs760732823), and 4950G > A (rs371441113) were assessed in 971 subjects. For each SNP, we randomly repeated approximately 10% of the PCR assays and evaluated concordance with DNA sequencing using an automatic sequencer (ABI3730 × 1 DNA analyzer; Applied Biosystems, Foster City, CA, USA). Concordance of these quality control samples was 100%. Details regarding the genotyping methodology and the *RNF213* SNP primer sequences were previously reported [21]. 

### 4.4. Statistical Analysis

The characteristics were expressed as mean ± standard deviation for continuous variables (age) and number (%) for categorical variables. Comparison of characteristics between groups were performed with independent t-test (continuous variables) or chi-squared test (categorical variables). *RNF213* SNP genotype frequencies were evaluated using the Hardy–Weinberg equilibrium test, which satisfied the Hardy–Weinberg equilibrium. To assess the relationships between *RNF213* variants and the presence of anterior/posterior ICASO, we calculated AOR and 95% CI using multivariate logistic regression analysis with the presence of anterior/posterior ICASO as the dependent variable. Regression models were adjusted for sex, age, and the presence of hypertension, DM, hyperlipidemia, atrial fibrillation, and ischemic stroke. The statistical analysis was performed with R software version 3.5.1 (The R Foundation for Statistical Computing, Vienna, Austria; http://www.R-project.org/). Linkage disequilibrium maps of *RNF213* variants were generated using Haploview version 4.2 (https://www.broadinstitute.org/haploview/haploview) [48]. Two-sided *p*-value <0.05 was considered statistically significant.

## 5. Conclusions

*RNF213* variants (4810G > A and 4950G > A) were significantly associated with anterior ICASO. Conversely, the *RNF213* 4863G > A variant was significantly associated with posterior ICASO. Our findings suggest a pathogenetic role of *RNF213* in ICASO. There is a clear need for further investigations elucidating the functional role of RNF213 to better understand the underlying pathogenesis for cerebrovascular disease.

## Figures and Tables

**Table 1:** Clinical characteristics of study subjects.

Variable	Total *n* = 1024	ICASO (-) *n* = 788	ICASO (+) *n* = 236	*p*-Value ^1^
Sex, male	481 (47.0)	353 (47.6)	128 (45.2)	0.535
Age, years	63.6 ± 11.4	62.5 ± 11.5	66.2 ± 10.4	<0.001
Medical history				
Hypertension	542 (52.9)	373 (50.3)	169 (59.7)	0.009
Diabetes mellitus	214 (20.9)	138 (18.6)	76 (26.9)	0.005
Hypercholesterolemia	280 (27.3)	195 (26.3)	85 (30.0)	0.265
Atrial fibrillation	59 (5.8)	38 (5.1)	21 (7.4)	0.208
Ischemic stroke	636 (62.1)	386 (52.1)	250 (88.3)	<0.001
ICASO in anterior cerebral circulation ^†^	236 (23.0)	-	236 (83.4)	-
ICASO in posterior cerebral circulation ^‡^	84 (8.2)	-	84 (29.7)	-

Note: Data are expressed as numbers (%) or mean ± standard deviation. ICASO, intracranial major artery stenosis/occlusion. ^1^ Comparison between ICASO (-) and ICASO (+). ^†^ Intracranial internal carotid artery, anterior cerebral artery, or middle cerebral artery. ^‡^ Intracranial vertebral artery, basilar artery, or posterior cerebral artery.

**Table 2:** Genotypes of *RNF213* variants.

Genotype	*RNF213* Single Nucleotide Polymorphism (RefSNP Number)			
	4448G > A (rs148731719)	4810G > A (rs112735431)	4863G > A (rs760732823)	4950G > A (rs371441113)
GG	826 (85.1)	983 (96.0)	755 (77.8)	839 (86.4)
GA	140 (14.4)	41 (4.0)	204 (21.0)	128 (13.2)
AA	5 (0.5)	0 (0)	12 (1.2)	4 (0.4)
*p*-value for test of HWE	>0.999	>0.999	0.7582	>0.999

Note: Data are expressed as numbers (%). HWE, Hardy–Weinberg equilibrium; *RNF213,* RING finger protein 213 gene.

**Table 3:** Genotype distribution of *RNF213* variants according to the presence of ICASO.

*RNF213* Polymorphism (RefSNP Number)	Genotype Frequency (GG/GA/AA)					
	ICASO (-)	ICASO (+)	Anterior ICASO (-)	Anterior ICASO (+)	Posterior ICASO (-)	Posterior ICASO (+)
4448G > A (rs148731719)	596/100/3	230/40/2	638/103/4	188/37/1	753/132/4	73/8/1
4810G > A (rs112735431)	716/25/0	267/16/1	762/26/0	221/15/1	703/37/0	80/4/1
4863G > A (rs760732823)	535/158/6	220/46/6	576/161/8	179/43/4	684/198/7	71/6/5
4950G > A (rs371441113)	613/83/3	226/45/1	655/86/4	184/42/0	767/119/3	72/9/1

Note: Data are expressed as numbers. ICASO, intracranial major artery stenosis/occlusion.

**Table 4:** Risk of ICASO according to genotype of *RNF213* variants.

*RNF213 Polymorphism*	Genotype	ICASO		Anterior ICASO		Posterior ICASO	
		AOR (95% CI)	*p*-Value	AOR (95% CI)	*p*-Value	AOR (95% CI)	*p*-Value
4448G > A (rs148731719)	GG	1.00 (ref)	-	1.00 (ref)	-	1.00 (ref)	-
	GA	1.21 (0.79–1.86)	0.388	1.43 (0.92–2.21)	0.109	0.74 (0.32–1.53)	0.454
	AA	1.51 (0.23–9.98)	0.671	0.71 (0.04–5.27)	0.769	2.32 (0.11–17.92)	0.473
	Dominant (GG vs. GA + AA)	1.22 (0.79–1.86)	0.356	1.40 (0.90–2.14)	0.131	0.80 (0.36–1.60)	0.562
	Recessive (GG + GA vs. AA)	1.46 (0.18–9.83)	0.693	0.67 (0.03–4.97)	0.732	2.41 (0.12–18.64)	0.451
4810G > A (rs112735431) ^2^	GG	1.00 (ref)	-	1.00 (ref)	-	1.00 (ref)	-
	GA	2.05 (0.98–4.21)	0.053	2.39 (1.14–4.87)	0.018	1.23 (0.35–3.35)	0.708
	AA	NA		NA		NA	
4863G > A (rs760732823)	GG	1.00 (ref)	-	1.00 (ref)	-	1.00 (ref)	-
	GA	0.69 (0.47–1.02)	0.067	0.86 (0.57–1.28)	0.467	0.29 (0.11–0.64) ^1^	0.005
	AA	2.96 (0.79–11.24)	0.104	1.66 (0.44–6.27)	0.453	8.55 (2.17–32.51) ^1^	0.002
	Dominant (GG vs. GA + AA)	0.76 (0.52–1.10)	0.157	0.90 (0.61–1.32)	0.588	0.52 (0.25–0.98) ^1^	0.043
	Recessive (GG + GA vs. AA)	3.19 (0.86–12.12)	0.081	1.72 (0.42–6.27)	0.425	10.32 (2.62–39.22) ^1^	<0.001
4950G > A (rs371441113)	GG	1.00 (ref)	-	1.00 (ref)	-	1.00 (ref)	-
	GA	1.43 (0.93–2.19)	0.100	1.71 (1.11–2.63) ^1^	0.015	0.82 (0.36–1.63)	0.589
	AA	0.49 (0.02–3.98)	0.542	NA		2.28 (0.11–19.34)	0.489
	Dominant (GG vs. GA + AA)	1.38 (0.90–2.10)	0.133	1.59 (1.03–2.44) ^1^	0.033	0.87 (0.40–1.70)	0.700
	Recessive (GG + GA vs. AA)	0.46 (0.02–3.74)	0.510	NA		2.35 (0.11–19.90)	0.473

Note: Data are adjusted odds ratios (AOR) and 95% confidence intervals (CI) derived from multivariate logistic regression models adjusting for sex, age, and presence of hypertension, diabetes mellitus, hypercholesterolemia, atrial fibrillation, or ischemic stroke. ICASO, intracranial major artery stenosis/occlusion; NA, not applicable (no cases in the arm). ^1^ *p*-value <0.05. ^2^ For *RNF213* 4810G > A, analysis for dominant and recessive models was omitted due to there being no subject with AA genotype.

**Table 5:** Haplotype analysis of *RNF213* variants for the presence of ICASO.

Haplotype	Frequency	ICASO		Anterior ICASO		Posterior ICASO	
*RNF213* 4448/4810/4863/4950		AOR (95% CI)	*p*-Value	AOR (95% CI)	*p*-Value	AOR (95% CI)	*p*-Value
GGGG	0.784	1.00 (ref)	-	1.00 (ref)	-	1.00 (ref)	-
AGGA	0.047	1.26 (0.76–2.10)	0.371	1.37 (0.81–2.32)	0.246	1.01 (0.46–2.24)	0.978
AGGG	0.024	1.02 (0.45–2.30)	0.964	1.41 (0.64–3.13)	0.398	0.31 (0.04–2.35)	0.258
GAGG	0.014	2.86 (1.15–7.11)	0.023	4.01 (1.63–9.87)	0.002	1.28 (0.33–4.97)	0.725
GGAG	0.104	0.87 (0.59–1.28)	0.472	0.95 (0.64–1.43)	0.811	0.88 (0.49–1.59)	0.680
Rare *	0.016	1.43 (0.48–4.28)	0.518	2.06 (0.69–6.12)	0.193	0.62 (0.08–4.88)	0.646

Note: Adjusted odds ratio (AOR) and 95% confidence interval (CI) were computed for each haplotype and compared to the most common haplotype (GGGG). Adjustments were made for sex, age, and the presence of hypertension, diabetes mellitus, hyperlipidemia, atrial fibrillation, or ischemic stroke. ICASO, intracranial major artery stenosis/occlusion. * Other rare combinations besides the above haplotypes.

## Abbreviations

AOR	Adjusted odds ratio
CI	Confidence interval
CTA	Computed tomography angiography
DM	Diabetes mellitus
HWE	Hardy–Weinberg equilibrium
MMD	Moyamoya disease
MRA	Magnetic resonance angiography
PCR–RFLP	Polymerase chain reaction–restriction fragment length polymorphism
*RNF213*	RING finger protein 213 gene
SNP	Single nucleotide polymorphism
